# Soft Matter under Pressure: Pushing Particle–Field Molecular Dynamics to the Isobaric Ensemble

**DOI:** 10.1021/acs.jcim.3c00186

**Published:** 2023-03-28

**Authors:** Samiran Sen, Morten Ledum, Sigbjørn Løland Bore, Michele Cascella

**Affiliations:** Hylleraas Centre for Quantum Molecular Sciences and Department of Chemistry, University of Oslo, P.O. Box 1033 Blindern, 0315 Oslo, Norway

## Abstract

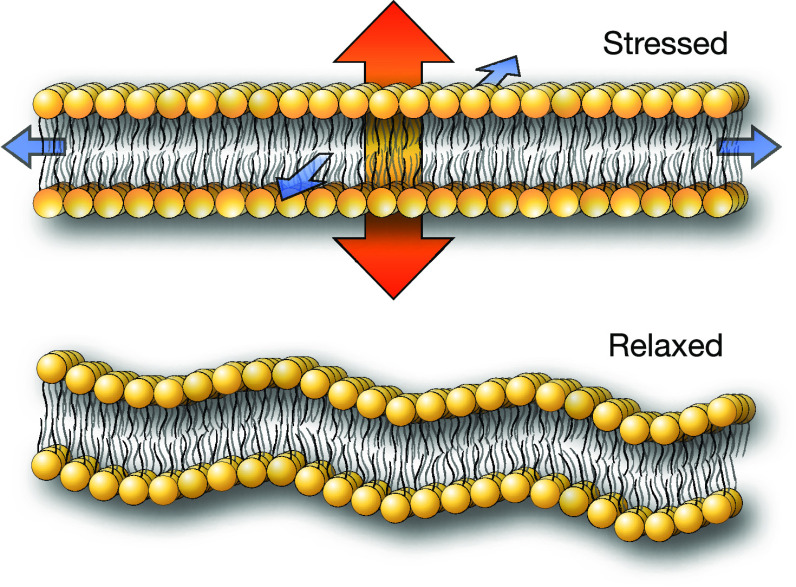

Hamiltonian hybrid particle–field molecular dynamics is a computationally efficient method to study large soft matter systems. In this work, we extend this approach to constant-pressure (NPT) simulations. We reformulate the calculation of internal pressure from the density field by taking into account the intrinsic spread of the particles in space, which naturally leads to a direct anisotropy in the pressure tensor. The anisotropic contribution is crucial for reliably describing the physics of systems under pressure, as demonstrated by a series of tests on analytical and monatomic model systems as well as realistic water/lipid biphasic systems. Using Bayesian optimization, we parametrize the field interactions of phospholipids to reproduce the structural properties of their lamellar phases, including area per lipid, and local density profiles. The resulting model excels in providing pressure profiles in qualitative agreement with all-atom modeling, and surface tension and area compressibility in quantitative agreement with experimental values, indicating the correct description of long-wavelength undulations in large membranes. Finally, we demonstrate that the model is capable of reproducing the formation of lipid droplets inside a lipid bilayer.

## Introduction

Biological membranes are a crucial component of all life forms. They compartmentalize cellular regions of strongly different chemical media while allowing selective passage of critical matter through them by means of their topological malleability.^1^ Their importance in life-sustaining roles is indisputable, and over the past two decades, molecular modeling of biological membranes has been actively pursued by scientific communities at large to better understand their complex roles in the diverse dynamics of living cells involving transport, growth, neural function, immunological response, signaling, and enzymatic activity.^2^ The obstacle faced in a complex quest such as this is broadly twofold: (i) many different chemical compositions of membranous structures exist in nature, further complicated by membrane–protein interactions,^3,4^ and (ii) structural dynamics like buckling and micellar and tubular formations occur at large characteristic length and time scales of hundreds of nanometers and several microseconds, respectively. Recent computational advances have turned the tables from studying small, simple bilayers over short time lengths to much larger near-realistic biomembranes over longer time lengths. This leap in computational accessibility implied the feasibility of using simulation approaches to better understand membrane properties at a molecular level of detail, which is intrinsically closed to available experimental approaches, like NMR and electron microscopy.^5^ The need for more accurate modeling of such systems has been looming ever since.

Over the last decades, different all-atom (AA) and united-atom (UA) force field parameters for lipids have been proposed.^6−10^ Comparative studies of force fields developed for membrane systems can be found, for example, in refs (11) and (12). Despite better models and increased computational resources, an atomic-level resolution of membrane systems for routine studies of thousands of lipid molecules over the microsecond time scale remains significantly costly. Coarse-grained (CG) approaches, adopting a lower-resolution description, alleviate this problem significantly and have been successful in describing a wide range of structural and dynamical properties for lipid systems.^5,13−16^ While CG methods reduce the number of particles for which forces have to be calculated, the general bottlenecks of AA methods, typically associated with frequent communication between parallel processors, persist. Hybrid particle–field (hPF) approaches offer an alternative to such direct CG particle–particle methods. hPF maintains the granular molecular representation of matter, substituting the interaction energy between bodies with external fields that depend on the density. Since the early *single chain in mean field* development within a Monte Carlo formalism by Müller and Smith,^17^ hPF has been applied to several soft matter systems, including coarse-grained copolymer films^18^ and polymer nanocomposites.^19^ Coupling to MD,^20^ hybrid particle–field methods have been successfully used to investigate entities like lipid bilayers,^21^ polyelectrolytes,^22−24^ and micelle morphology.^25^ Also, Qi and Schmid^26^ have used the hybrid nature of the method dynamically to study diffusion through Brownian motion coupled with a local density functional theory in soft condensed matter systems. A further description of past research in hPF can be found in ref (27).

For realistic simulations of membranes, constant-pressure simulations that probe membranes exposed to external stress are necessary. NPT simulations within the hybrid particle–field formalism have proven difficult. The lack of proper treatment of the pressure tensor is problematic for an accurate description of multiphase systems, where structural inhomogeneity yields to a systematic imbalance between the components of the internal pressure, which affect crucial interfacial properties like surface tension. After a first attempt to compute the scalar pressure within the hybrid particle–field formalism,^28^ in 2017 Ting and Müller^29^ devised a self-consistent field theory to obtain conjugate potential and volume fraction fields wherefrom they derived a local stress profile expression for soft interfacial systems. In 2020, Bore et al.^30^ extended the hPF formalism to perform constant-pressure simulations by determination of the full pressure tensor. A key observation of this work was that the available hPF-MD models required an additional energy functional based on square gradients of particle densities to produce anisotropic pressure contributions and to yield a realistic description of interfacial properties. However, in simulations of lipid bilayers, the model reported an excessive rigidity, with an area compressibility two orders of magnitude higher than in experiments.

Starting from seeding work by Theodorou and co-workers,^31,32^ two of us recently introduced a Hamiltonian hybrid particle–field (HhPF) approach.^33^ Contrary to previous derivations of hybrid particle–field models that were based on statistical field theory and saddle-point approximation arguments, our derivation was based on filtered densities and a particle-mesh formalism that rigorously derives the force on particles for any given interaction potential. We established that the physics described by HhPF is formally equivalent to that of particle models interacting by two-body potentials,^33^ and thus, it must be able to reproduce all molecular properties accessible by such models, including anisotropic pressure.^34^ Motivated by these considerations, here we pursue the determination of anisotropic pressure within the HhPF formalism, showing how this approach is able to achieve realistic simulations of soft matter systems exposed to external stress.

## Theory

### Hamiltonian Hybrid Particle–Field

For a system of *M* molecules, with the *i*th molecule containing *N*_*i*_ particles, we consider the following Hamiltonian:^33^
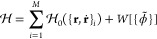
1
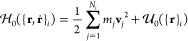
2where  is the Hamiltonian for the *i*th molecule,  is the internal potential energy of a molecule, containing only the typical molecular mechanics bonded terms, and *W*[{ϕ̃}] is the interaction energy functional, dependent on the densities of the particles {ϕ̃}, modeling nonbonded interactions. *W*[{ϕ̃}] is expressed as an integral of the local interaction energy *w*:
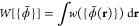
3The density functions are numerically obtained from the instantaneous particle positions by standard particle-mesh operations.^35^ Following ref (33), this involves the convolution of a cloud-in-cell assignment function on a regular grid, *P*(**r** – **r**_*i*_), with a Gaussian filter  that defines the density spread associated with each particle type:
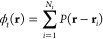
4
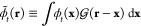
5where *N*_*t*_ is the number of particles of type *t* and ϕ̃_*t*_(**r**) is the density of that type.

Following Bore et al.,^30^ we opt for an interaction energy functional of the form
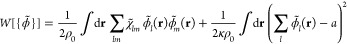
6where χ̃_*lm*_ is the mixing interaction parameter between particle densities of types *l* and *m*, ρ_0_ is an intrinsic parameter corresponding to the specific volume of a coarse-grained particle, κ is an incompressibility parameter that controls the fluctuations of the density locally, and *a* is a free parameter that can be tuned to calibrate the correct average density at the target temperature and pressure of interest.

The force on a particle placed at **r**_*i*_ can be obtained by the direct spatial derivative of this interaction energy functional as
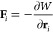
7The above can be recast, as worked out in ref (33), into the form
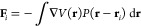
8where , called the *external potential*, and its gradient are obtained numerically by fast Fourier transform (FFT) operations.^33^

### Derivation of Pressure

In a molecular system, the internal pressure can be calculated from its internal energy components as^36^
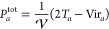
9
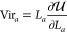
10
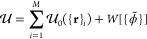
11where  is the volume of the system, Vir is the virial of the total interaction energy  comprising both bonded () and field terms, and, *L*_*a*_ and *T*_*a*_ are the length of the box and kinetic energy in the *a*th Cartesian direction, respectively. *T*_*a*_ is simply *T̅*/3, where *T̅* = 3*k*_B_*T*/2 is the total kinetic energy, with Boltzmann constant *k*_B_ and temperature *T*. Here we show the essence of the derivation of the internal pressure, while further mathematical details are provided in SI: Field Pressure: A Complete Derivation in the Supporting Information.

Inserting eq 11 into eq 10 and the result into eq 9, we obtain
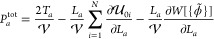
12The first two terms correspond to standard contributions to the pressure from the kinetic energy and the molecular bonded terms, respectively. We introduce a new variable *V̅*_*t*_(**r**) that we call a *filtered potential*:
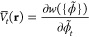
13A more explicit expression for *V̅* is given in SI: Expansion of the Filtered Potential. Then, the third term of eq 12 is expanded using the chain rule across the filtered densities to obtain
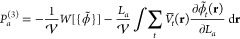
14where the index *t* runs over all particle types. The derivative in the second term has dependencies on both the assignment function and the filter. Developing it in Fourier space (for details, see SI: Field Pressure: A Complete Derivation) yields
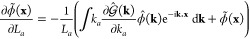
15For a Gaussian filter with spread σ,
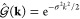
16
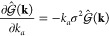
17Considering that
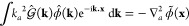
18we obtain
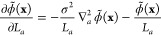
19Thus, we obtain an anisotropic term in eq 19 in the form of the Laplacian of particle densities, which was not present in previous formulations.^30^ Putting eq 19 into eq 14 with ϕ̃ as ϕ̃_*t*_, we get the final pressure equation due to the density field:
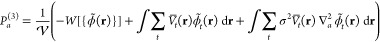
20

## Methods

### Computational Details

#### Pressure Implementation and Simulation Details

All constant-pressure simulations were carried out in the NPT canonical ensemble at a pressure of 1 bar and at room temperature in the range of 300–323 K. The temperature was controlled by a canonical sampling by velocity rescale (CSVR) thermostat^37^ with a typical coupling time constant of τ = 0.1 ps. External pressure was controlled using a Berendsen barostat.^38^ We also implemented a stochastic cell rescaling (SCR) barostat,^37^ which guarantees correct statistical sampling of the isothermal–isobaric ensemble and was used to validate some of the results presented here. The compressibility parameter was set to the isothermal compressibility of water (β = 4.6 × 10^–5^ bar^–1^), and a coupling time constant τ_*P*_ was chosen from a range of 0.1–1.0 ps such that the pressure coupling always remains stronger than the thermostat coupling. We used an rRESPA multiple time step algorithm where the slowly evolving field forces are integrated at a lower frequency while the rapidly evolving bonded forces are integrated at a higher frequency.^39^ In all HhPF-NPT runs reported in this paper, the two time steps used are Δ*t*_1_ = 0.3 ps and Δ*t*_2_ = 0.03 ps, respectively. More details can be found in ref (39). Our HhPF approach was implemented into the HylleraasMD (HyMD) modeling package.^39−41^ More on this and the implementation of the barostat can be found in SI: Pressure Implementation.

#### System Details

For all systems discussed in the Results, details of the system setup and simulation specification are listed in Table 1. For the *a* parametrization, the homogeneous system was run for 1 ns with different pairs of (κ, σ). The calibrated *a* obtained was kept unchanged for all other systems run under NPT conditions. The multiphase system comprised two types of particles that were designed to be immiscible by using a large positive interaction parameter χ̃ = 36 in eq 6. Self-interactions in HhPF were administered by the second term in the same Hamiltonian expression with a compressibility factor κ of 0.05 mol kJ^–1^. The CG mapping used for the dipalmitoylphosphatidylcholine (DPPC) systems is described in SI: Coarse-Grained Mapping. For the pure DPPC bilayer systems (DPPC2_A and DPPC2_B), simulations were repeated using both standard hPF^42^ and machine-learned (ML) interaction χ̃ parameters, while for the DPPC–triglyceride systems (DPPC4_TG1 and DPPC4_TG2) and the large DPPC system (DPPC3) only ML parameters were used. The parameters are listed in Table S1. These runs were performed for 100 ns after an initial relaxation.

**Table 1:** System Details

system	no. of molecules	no. of solvent molecules^a^	box (*x*, *y*, *z*) [nm]^b^	method	*T* [K]^c^
homogeneous	1171	–	(5.2, 5.2, 5.2)	HhPF	300
multiphase	5083 A + 5472 B	–	(10.6, 10.6, 10.6)	HhPF	300
DPPC1	318 DPPC	14784	(9.9, 9.9, 10.0)	AA	300
DPPC2_A	528 DPPC	14000	(13.0, 13.0, 14.0)	HhPF	323
DPPC2_B	528 DPPC	14000	(11.9, 11.9, 17.4)	HhPF	323
DPPC3	31800 DPPC	1267766	(100.0, 100.0, 20.0)	HhPF	323
DPPC4_TG1	1024 DPPC + 20 TG	29308	(17.9, 17.9, 16.0)	HhPF	323
DPPC4_TG2	1024 DPPC + 56 TG	28086	(17.9, 17.9, 16.0)	HhPF	323

a Water was used as the solvent for all of the simulations. The number reported for AA runs is the number of TIP3P water molecules, and for HhPF runs it is the number of coarse-grained particles with a 4:1 water mapping.

b Initial box dimensions at which the system was set up.

c All simulations were run in the NPT ensemble with *P* = 1 bar.

#### Area Compressibility

For the DPPC2_B system, the surface tension γ was calculated as
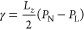
21where *L*_*z*_ is the height of the box normal to the bilayer and *P*_N_ and *P*_L_ are the internal pressures along the normal and lateral directions, respectively. By progressively increasing the tension on the bilayer by changing the external lateral pressure in the range of 0–50 bar, the system was sampled at an increasing area per lipid *A*_L_. The area compressibility *K̅*, often referred to as the bilayer modulus, was then estimated from a linear least-squares fit of the following expression:^43^
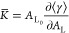
22where *A*_L_0__ is the area per lipid measured under tensionless conditions (γ = 0) and the average ⟨γ⟩ is taken over each constant-area simulation at area per lipid *A*_L_. A detailed description of the method is given in SI: Constant Area Simulations.

#### Thermal Undulations

Any more than a 20 nm long patch of a bilayer is considered valid to apply continuum theory to extract the frequency modes of thermal undulations.^44^ The large DPPC3 system was used to study characteristic undulations in simple membrane systems by extracting the frequency modes of fluctuations assuming a continuum model.^45^ The membrane surfaces were traced out from the positions of the G particles of the CG representation of DPPC (see SI: Coarse-Grained Mapping) according to^45^
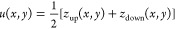
23where the membrane is placed in the *xy* plane and *z*_up_ and *z*_down_ are the *z* coordinates of the G particles in the upper and lower monolayers, respectively. The corresponding fluctuation spectrum was obtained in Fourier space under tensionless conditions as
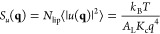
24where *N*_lip_ is the number of lipids per monolayer, *u*(**q**) is the Fourier transform of the real-space membrane surface *u*(*x*, *y*), **q** = (*q*_*x*_, *q*_*y*_) is the wave vector, and *K*_c_ is the bending modulus. The method of analysis is similar to that in ref (44). The fluctuation spectrum was fitted with a straight line for the first 11 *q* values, where approximately *q* < 1 nm^–1^, corresponding to the *q* range where microscopic protrusions are considered absent.^44^

All of the DPPC bilayer systems were built in CHARMM-GUI,^46−48^ and all-atom NPT runs of the pure DPPC system were performed using the CHARMM36^49,50^ force field on GROMACS.^51^ The all-atom DPPC systems were coarse-grained with a Martini mapping^52^ with a 4:1 solvent mapping. The masses of all coarse-grained particles were treated as equal.

### Parameter Optimization

The χ̃ parameters in eq 6 were optimized by a gradient-free Bayesian optimization (BO) protocol^53^ using an upper confidence bound acquisition function^54,55^ as in ref (42). The target fitness (η) was defined as the sum of the symmetric mean absolute percentage error (SMAPE)^56^ of the HhPF number density profiles with respect to reference all-atom data from simulations using the CHARMM36 force field^49,50^ and the squared error of *A*_L_ compared to the reference literature value of 0.63 nm^2^:^57^
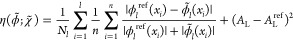
25where *N*_*l*_ denotes the number of particle types and *x*_*i*_ are discretized distances along the bilayer normal (histogram bin locations). The reference bilayer number density profile for species *l* is represented by ϕ_*l*_^ref^(*x*).

A simple batch-parallel BO approach was employed wherein numerous optimization procedures are executed concurrently, with full data sharing between each optimization instance after each sampling. A different exploration–exploitation parameter β was used for each instance of the BO algorithm, ranging from 10^–3^ (heavily favoring exploitation) to 10^3^ (heavily favoring exploration). For each parameter set, eight simulations were run, and the fitness was averaged across them. The best set of optimized χ̃ parameters were chosen from among a few shortlisted sets on the basis of SMAPE. A more detailed description of the methodology is described in SI: Machine Learning with Bayesian Optimization.

## Results

### Homogeneous System

#### Calibration of the Equation of State Parameter *a*

**Figure 1:**
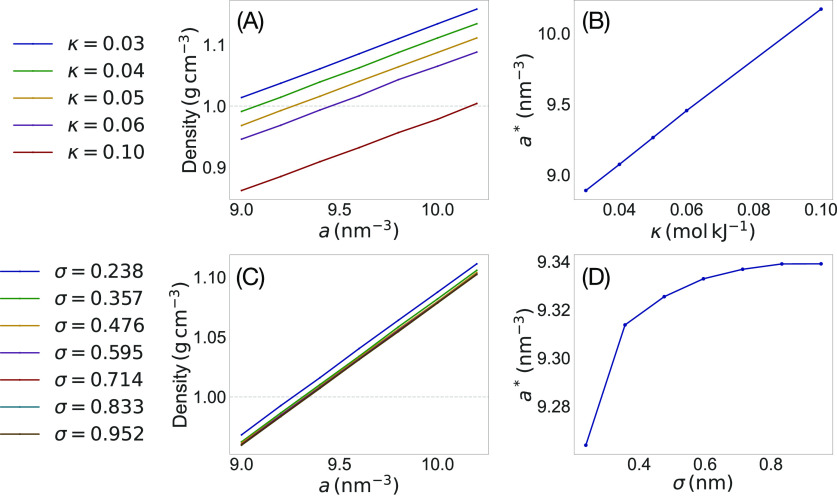
Calibration of the *a* parameter to obtain the natural water density of 1 g cm^–3^ at room temperature for (A, B) different κ parameters with σ = 0.238 nm and (C, D) different σ parameters with κ = 0.05 mol kJ^–1^. The asterisk (*) against *a* denotes the values of *a* for which the natural density of water was obtained.

The parameter *a* in eq 6 was calibrated for different values of the compressibility κ and particle spreads σ to reproduce the experimental density of water, as shown in Figure 1. From the top-right plot, we can see that *a* shows a linear dependence on the system compressibility, in line with what was reported in ref (30). The dependence of *a* on the particle spread is strongly linear at first because the overlaps of density increase with increasing σ in this region. It levels off for larger values, corresponding to the saturation of the particle overlap signal. Unless stated otherwise, for best consistency with past literature data, we chose the pair κ = 0.05 mol kJ^–1^, *a* = 9.21 nm^–3^ for all of our simulations.

### Multiphase Systems

#### Surface Tension

The field pressure (eq 20) describes the appearance of anisotropic pressure components proportional to the Laplacian of the density distributions. While this term is necessarily negligible within homogeneous moieties, it is expected to produce a finite pressure imbalance at interfaces, which are characterized by spatially well-organized density variations.

**Figure 2:**
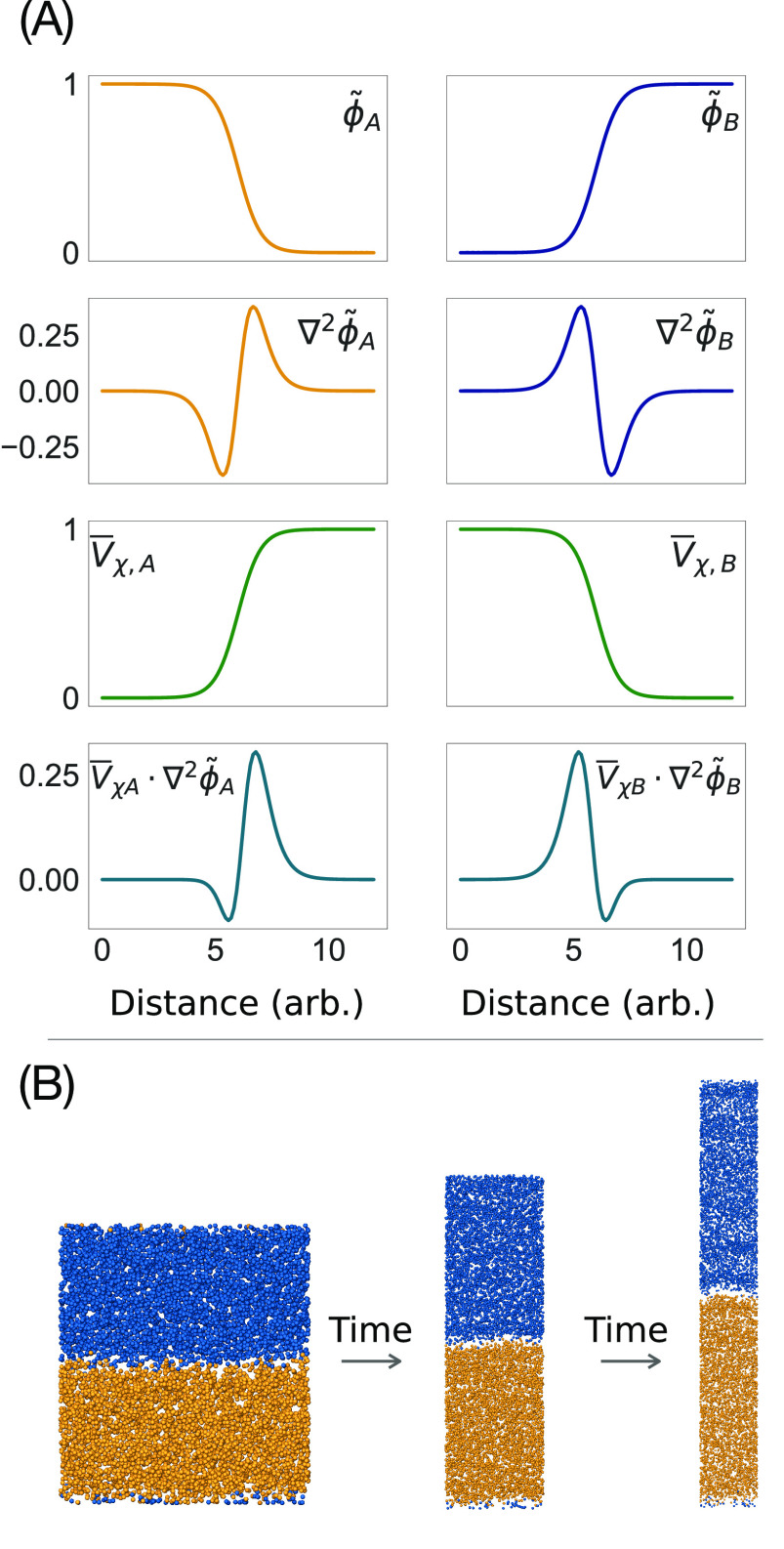
(A) Density and derivative plots for a binary phase-separated mixture of particles of type A (left panel) and B (right panel). From top: (i) density; (ii) Laplacian of density; (iii) filtered potential; (iv) product of (ii) and (iii). (B) Formation of a capillary from the expansion of a binary phase-separating mixture.

Figure 2A shows the relevant quantities entering into the calculation of the anisotropic pressure term for an ideal biphasic system described by analytic sigmoidal distributions (as described in SI: Analytic Study of Biphasic System) along the direction normal to the surface. Clearly, the model predicts the appearance of an extra positive pressure along the normal direction, correctly describing the presence of surface tension. Figure 2B describes the dynamic behavior of the corresponding numerical model of the same biphasic system, formed by two monatomic nonmiscible fluids (χ̃_AB_ = 36) during HhPF NPT simulations. As predicted by the analytic model, the tension promotes the reduction of the interfacial area, resulting in a progressive expansion of the simulation box in the normal direction, leading to the formation of a capillary.

### Membrane System

Having verified that the anisotropic pressure term correctly describes the qualitative pressure imbalance at model interfaces, we explored its behavior in realistic representations of interfacial systems, namely, phospholipid bilayers.

**Figure 3:**
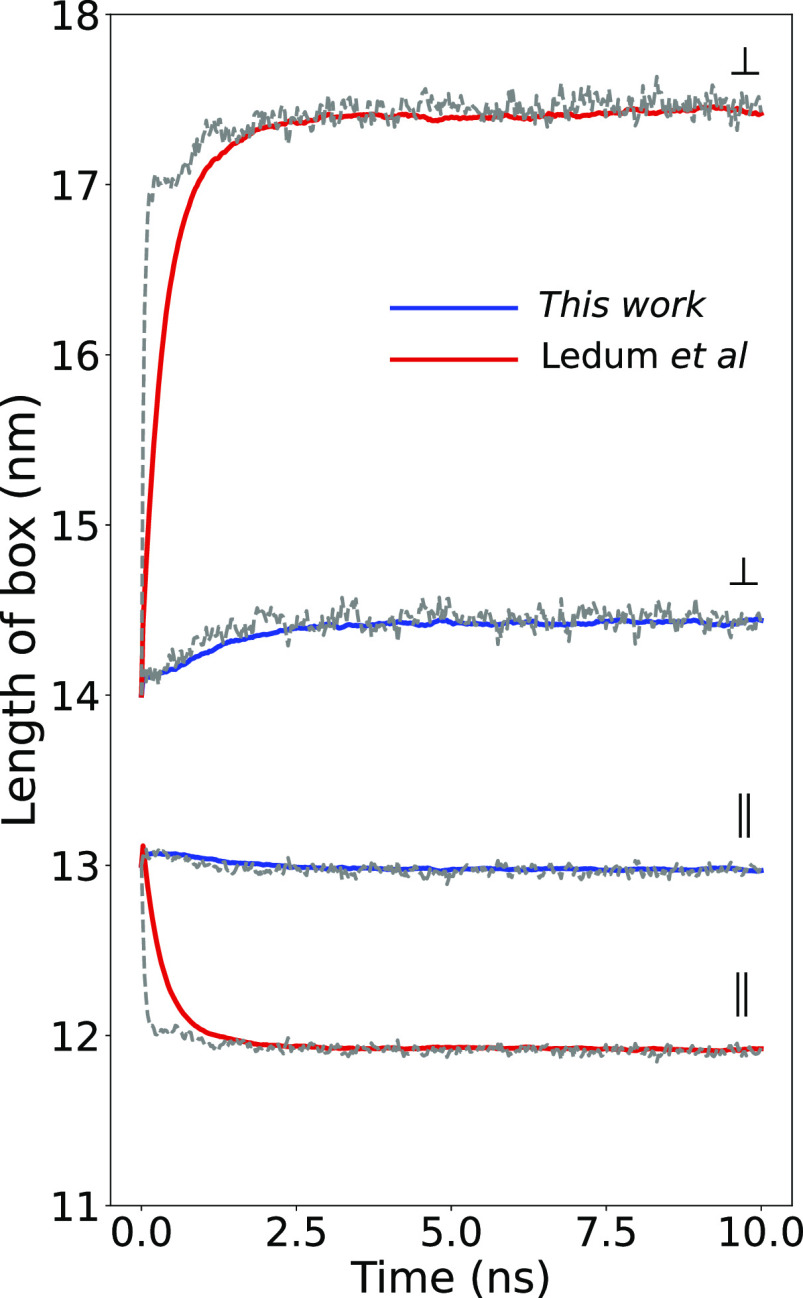
Relaxation of a DPPC bilayer under NPT conditions in the directions normal to (⊥) and along (∥) the membrane plane using literature NVT-BO-optimized χ̃ parameters from Ledum et al.^42^ (red) and the current NPT-BO-optimized χ̃ parameters (blue). The system was simulated with the Berendsen barostat^38^ (solid lines) as well as the SCR barostat^37^ (gray/dashed lines).

In an initial set of simulations, we employed literature parameters from ref (42) to describe intermolecular forces. As shown in Figure 3, after an initial relaxation, the edges of the periodic simulation box equilibrate to lengths of *L*_*x*_ = *L*_*y*_ = 11.92 nm, *L*_*z*_ = 17.42 nm. The corresponding tensionless DPPC bilayer globally retains the initial lamellar structure. Specifically, the lateral density profiles are in close resemblance with the reference NVT conditions on which the χ̃ parameters were optimized. On the contrary, the box relaxation in the initial phase of the NPT simulation produced a reduction in the total surface of the bilayer, resulting in an average ⟨*A*_L_⟩ = 0.54 nm^2^, which is 18.5% smaller than what is reported in the literature for DPPC, listed in Table 2. Other literature χ̃ parameters based on estimates from Flory–Huggins mixing rules and that have been extensively used in previous hPF studies^21,30^ yielded a practically identical outcome under NPT conditions. That NVT-optimized parameters produce a smaller *A*_L_ can be understood with the fact that they were trained to reproduce the density profile of flat bilayers sampled over relatively small periodic boxes. Thus, calibration of parameters producing marginal surface tension, not evaluated during optimization, could facilitate the formation of flat moieties, resulting in a better fit to the reference data but poor transferability to the NPT ensemble.

**Table 2:** Comparison of Mechanical Properties of DPPC with the Literature: *T*, Temperature; *A*_L_, Area per Lipid; *K̅*, Area Compressibility; *K*_c_, Bending Modulus

		*T* (K)	*A*_L_ (nm^2^)	*K̅* (mN m^–1^)	*K*_c_ (10^–20^ J)
Experiment	Kucerka et al.^59^	323	0.630	231	6.7
	Petrache, Dodd, and Brown^60^	323	0.633	–	–
AA	Waheed and Edholm^61^	323	0.644	348	6.1
	Levine et al.^62^	323	0.629	210	15.6
hPF	Bore et al.^30^	325	0.64	22000	–
HhPF	this work	323	0.636	225	3.21

**Figure 4:**
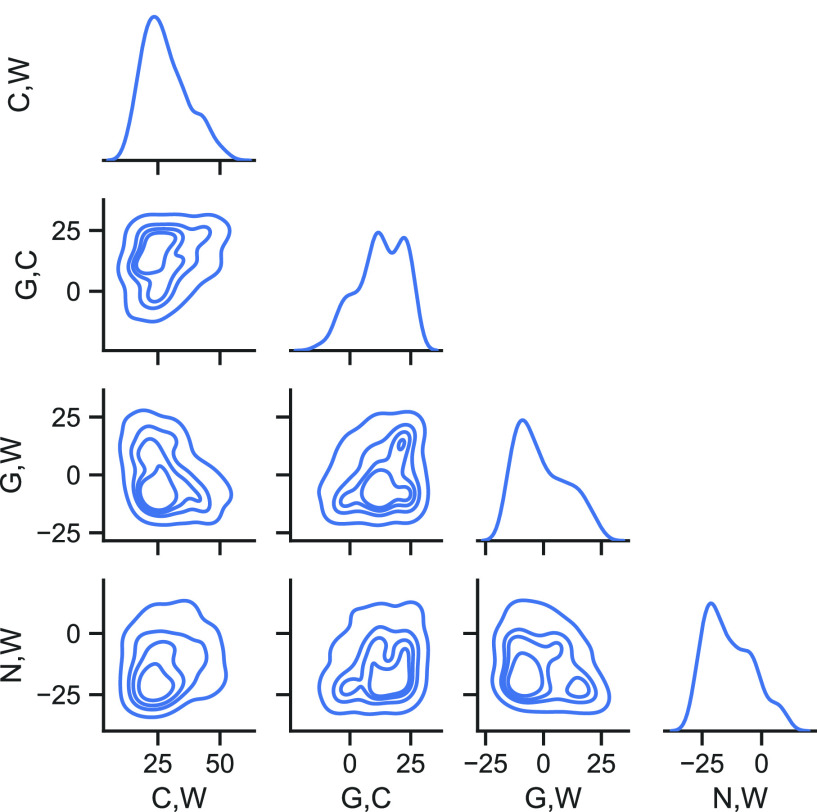
Correlation contours showing the χ̃ parameter space explored by BO. Only the four most important pair interactions in a DPPC bilayer (DPPC2_B system of Table 1) are shown. Across the diagonal are the density distributions of the sampled points for each χ̃. All χ̃ values are in kJ mol^–1^.

To take into account this issue, we reoptimized the χ̃ parameter set by Bayesian optimization in the NPT ensemble, this time targeting both the lateral density profiles and the *A*_L_ from all-atom models (see Methods). Figure 4 shows the region in the χ̃ parameter space that BO explores to minimize the fitness function. Dense and sparse contour regions, as well as the peaked density distributions of each χ̃ parameter along the diagonal, demonstrate the role of BO in effectively limiting the search in a subspace of the otherwise large and multidimensional parameter space. The NPT-BO-optimized parameters are listed in SI: Machine Learning with Bayesian Optimization. Relaxation of the system (DPPC2_A) using the NPT-BO-optimized parameters compared to the relaxation with literature parameters (Figure 3) shows a quick convergence to lengths *L*_*x*_ = *L*_*y*_ = 12.96 nm, *L*_*z*_ = 14.44 nm, corresponding to the correct *A*_L_ of 0.636 nm^2^ (Table 2).

**Figure 5:**
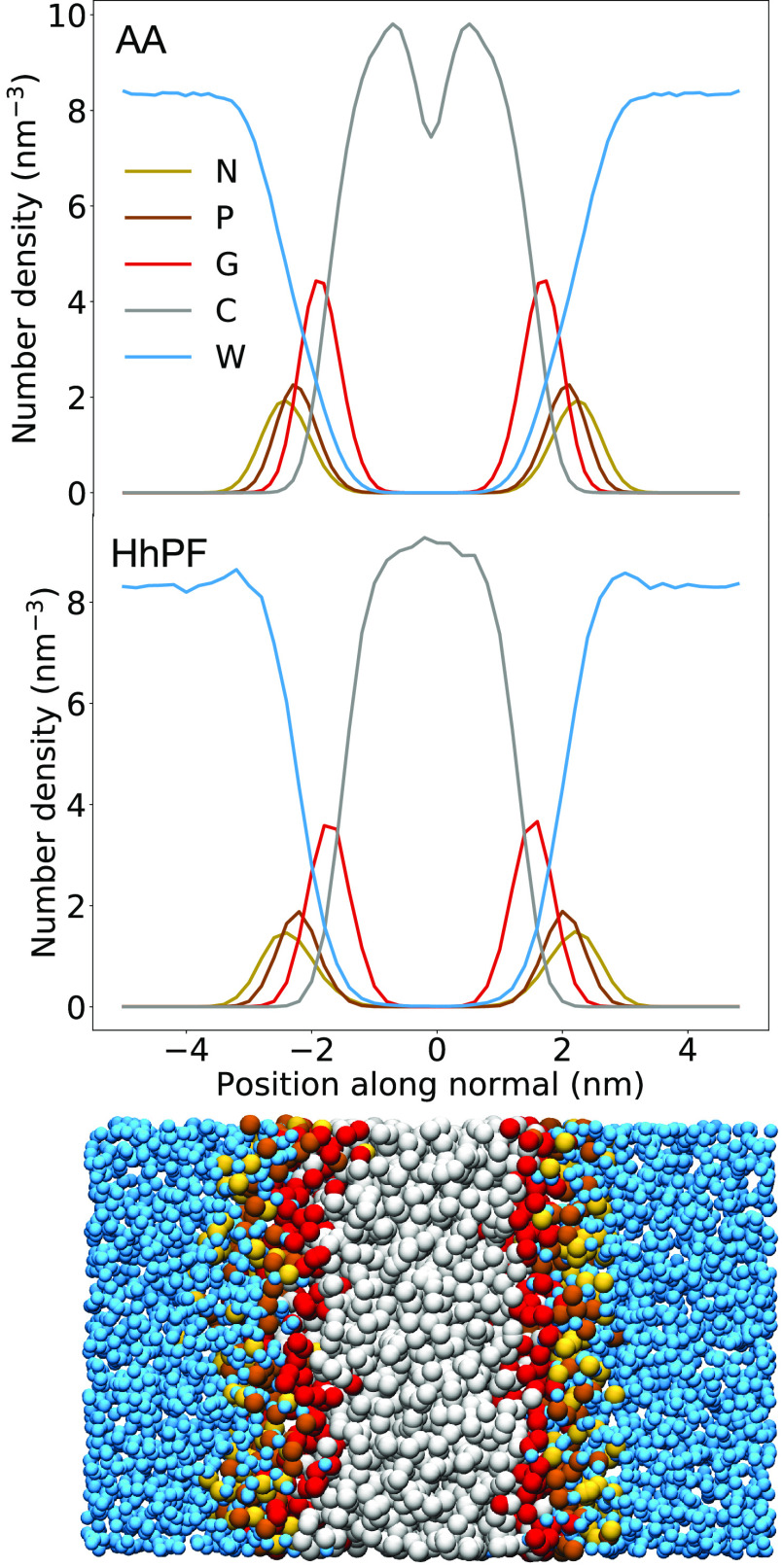
Local density profiles of each DPPC CG particle type from (top) an AA NPT run for reference, coarse-grained post-MD for comparison and (middle) from an HhPF NPT run. (bottom) Representation of a system of 528 DPPC molecules in a DPPC bilayer in water. Some bulk water and lipids have been removed for clarity.

Figure 5 shows the comparison between the density profiles obtained from target all-atom simulations and from HhPF-NPT simulations using the set of BO-optimized χ̃ parameters. The NPT optimizations produced both qualitative and quantitative agreement with the reference system, the only significant difference being associated with the lack of the density dip separating the lipid tails at the center of the membrane. Importantly, the density profiles show even better agreement with respect to the reference compared with previous NVT optimizations by BO,^42^ especially in an improved reproduction of the water penetration into the glycerol layer.

**Figure 6:**
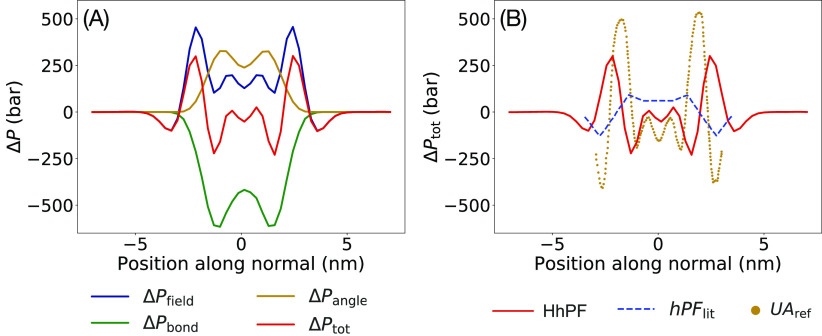
(A) Contribution to pressure differences between normal (N) and lateral (L) directions, denoted by Δ, from pressures due to the field (*P*_field_), two-particle bonds (*P*_bond_), three-particle bonds (*P*_angle_) and the total sum (*P*_tot_). Notation: Δ*P*_*t*_ = *P*_*t*_^*N*^ – *P*_*t*_^*L*^, where *t* = field, bond, angle, total. (B) Comparison of Δ*P*_tot_ across a membrane with HhPF in HyMD (solid red line), standard hPF (dashed blue line), and the united atom profile by Lindahl and Edholm^58^ (dotted gold line).

We also looked into the lateral profiles of internal pressure differences across the membrane. Figure 6A shows the contributions of the different potential energy terms to the local pressure profile along the axis normal to the membrane plane. The pressure components show a strong anisotropy due to the field components mostly at the lipid–water interface. Considering also the tensor components due to the bonded interactions, we obtain the characteristic modulation of the tension with negative contributions from the hydrophobic region and positive contributions at the level of the polar heads. Comparison with the reference united atom (UA) study by Lindahl and Edholm,^58^ shown in Figure 6B, indicates a very good quantitative agreement, in particular with HhPF data almost exactly reproducing pressure anisotropy modulations in the inner region of the membrane. The quantitative agreement decreases in the region corresponding to the polar heads and to the interfacial water, likely due to the unavoidable formation of fewer solvation structures than in fine-grained models. Most importantly, HhPF-NPT simulations reveal a remarkable improvement in the local pressure profiles compared to past hPF-NPT models,^30^ shown in the same Figure 6B, where only rough qualitative agreement with UA data could be achieved. We also note how HhPF is capable of reproducing with good accuracy a fine-grained estimate of the area compressibility, while equilibrated DPPC membranes from past hPF models showed excessive rigidity, with an area compressibility roughly two orders of magnitude higher than expected.^30^ The results are summarized in Table 2.

### Membrane Fluctuations

**Figure 7:**
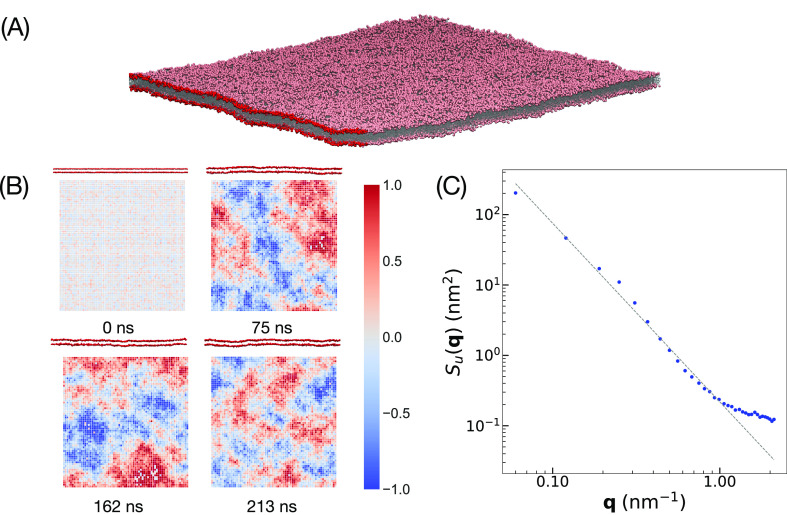
(A) Snapshot of a large DPPC bilayer with curvature. One edge has been brightened to highlight the curvature. (B) Side-view snapshots of the bilayer, with the corresponding heat maps of the positions of the G-type particles to show the undulations in the membrane, across NPT runtimes of 0, 75, 162, and 213 ns. (C) Undulation spectrum *S*_*u*_(**q**) as a function of the wave vector **q**. The first 11 small **q** values are fitted with a straight line, shown with a dotted line. Axes are in log scale.

Having demonstrated that the HhPF model for DPPC showcases accurate structural and mechanical properties, we further extended our investigation to its capability to capture intrinsic membrane undulations under tensionless conditions. As DPPC produces very flat bilayers, proper thermal undulations can be captured only by simulating relatively large patches in periodic boundary conditions. Lindahl and Edholm^58^ were among the first to directly observe fluctuations down to 0.3 nm^–1^ in the size-limiting case, which was extended later by Brandt, Braun, and co-workers^44^ to 0.1 nm^–1^. HhPF allows us to access large enough systems, and in this case we opted for a 100 nm × 100 nm periodic bilayer starting from a perfectly flat configuration and could detect natural thermal fluctuations as expected. A representative snapshot is shown in Figure 7A. Typical heat maps, plotted by tracing out an approximated membrane surface according to a Helfrich-type continuum model^45,63^ using eq 24, are shown in Figure 7B at a few specific timeframes. The fluctuation spectrum calculated from these maps is shown in Figure 7C, and the bending modulus of rigidity obtained from it was 3.21 × 10^–20^ J, which is in the correct order of magnitude of experimentally observed values (Table 2).

### Phase Separation

As a final test, we explored the capability of HhPF-NPT simulations to reproduce membrane relaxation associated with concentration-induced phase separation. For this, we considered a DPPC bilayer formed by 1024 lipid molecules into which a different number of triglyceride (TG) molecules were dissolved.

**Figure 8:**
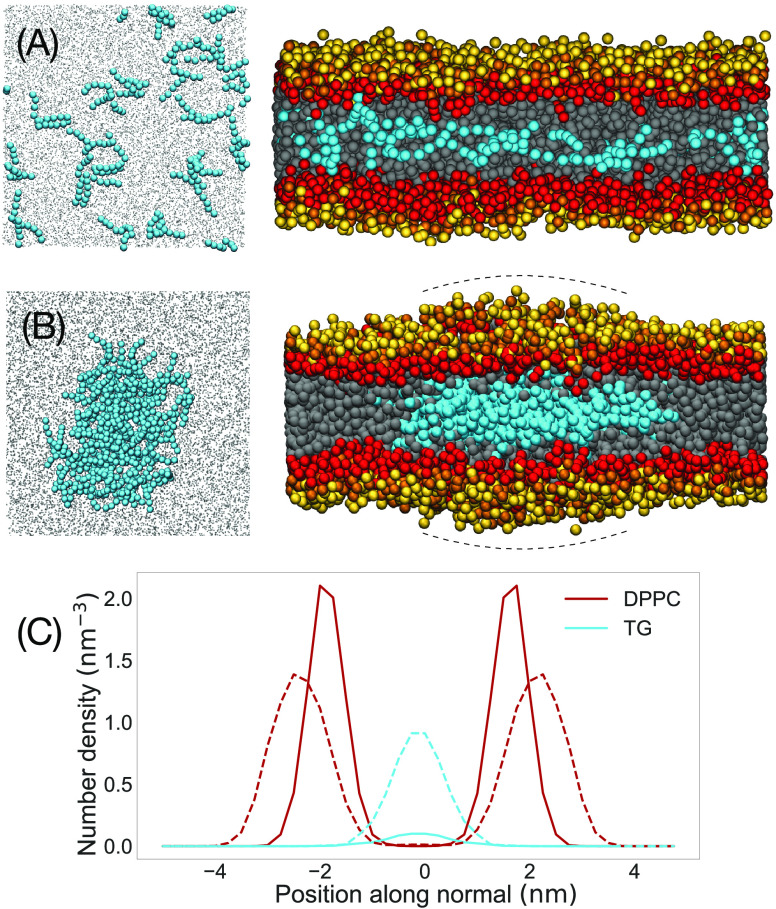
Triglycerides (TGs) inside a DPPC lipid bilayer. (A) Lower concentration of TGs in the dispersed state. (B) Higher concentration of TGs forming phase-separated lipid blisters facilitated by a bulging membrane, emphasized by curved dashed lines. In (A) and (B), the left panels are the top views and the right panels are the side views. The TG particles in the interior of the bilayer are in cyan amidst the carbon tails of DPPC in gray. Lipid headgroups are colored as in Figure 5. (C) Normal density profiles of DPPC and TG. The DPPC profile is represented by the G particle profile in the coarse-grained scheme. The TG profile is scaled up by a factor of 2 for clarity. Solid and dotted lines are the particle number densities corresponding to (A) and (B), respectively.

Figure 8A,B shows representative configurations after 100 ns of relaxation for the two 20:1024 and 56:1024 TG:DPPC concentrations that we considered, which lie below and above the 5% interfacial solubility limit of TG in DPPC. At low concentrations, we observe sequestered TGs that dissolve into the inner core of the bilayer. At higher concentrations, we observe the phase separation of TG, rapidly occurring within the 5 ns of simulations. TGs aggregate into an oblate ellipsoidal blister contained by the two DPPC monolayers. The rearrangement of the structure to accommodate the TG lens is associated with a relaxation of the membrane plane, narrowing it laterally by 8%. This is biologically expected and similar to TG-rich domains observed in ^1^H NMR spectra of a suspension of cancer cells.^64^Figure 8C shows the density distributions of DPPC and TG normal to the membrane plane in both cases of high and low TG concentrations. Our simulations also agree qualitatively well with CG simulations using the MARTINI model reported in ref (65), where the increase in concentration of TG is associated with a phase separation with the formation of a TG blister in the middle of the phospholipid bilayer.

## Conclusion and Outlook

We have presented a formulation of pressure within the Hamiltonian hybrid particle–field (HhPF) method of molecular dynamics by taking into account the intrinsic spread of each particle. We have implemented this in the present HhPF code, HyMD,^41^ making it now feasible to run simulations under constant-pressure conditions (NPT). We have shown that the very construct of the Hamiltonian approach, unlike previously understood, is adequate to describe the anisotropic components of the pressure, which are crucial for interfacial systems, without the need to introduce additional model energy terms. We applied the implementation to simple membrane bilayer systems, and using parameters that were optimized through machine learning we could produce density profiles, area per lipid, and also area compressibility in good agreement with experiment and united-atom studies. Not only were these global quantities accurate, but also the local variation of pressure across a membrane matched well with united atom references. In particular, automated parametrization by BO targeting lateral density profiles and the area per lipid yielded a model that has excellent predictivity for other quantities like pressure profiles and area compressibility, with overall agreements with the experimental values that are competitive with those from more accurate united atom references. For the first time within HhPF, to our knowledge, we evidenced the emergence of natural thermal fluctuations in a large membrane bilayer. We also briefly explored the phase separation of triglycerides in the interior of a membrane bilayer.

At this juncture, a natural next step is to use the constant-pressure implementation on chemically similar moieties, like other lipids with unsaturated tails, to test the transferability of the ML interaction parameters, as well as on lipids with saturated and polyunsaturated tails with different polar heads like phosphocholine and phosphoethanolamine,^3^ to build a library of HhPF parameters that can reproduce properties such as those shown in the case of pure DPPC in this work. Furthermore, by straightforwardly implementing electrostatic interactions using the Ewald summation, as has already been done in the previous release of HyMD,^39^ from the NVT ensemble into the NPT ensemble, studies on charged systems like peripheral and integral proteins embedded into membrane bilayers^5^ or membranes in ionic media can be conducted.

The successful initial test of observing a lipid blister formation in the interior of membrane systems opens many doors of interest. For example, the exact mechanism of lipid droplet (LD) biogenesis is unknown and has been under the scrutiny of the LD research community for years now. In 2017, on the basis of evidence from structural changes in lipid bilayers that they observed in united-atom MD simulations, Bacle et al.^66^ suggested a mechanism wherein LD formation can start from triglycerides clustering together between the leaflets of the endoplasmic reticulum. The fact that the present HhPF study was able to reproduce a similar phenomenon inside a bilayer not only verifies the hypothesized mechanism but also significantly extends the system-size- and simulation-time-dependent confines.

With the pressure calculations and barostat in place, the horizons of HhPF have expanded greatly and are now reaching a level of maturity to be reliably applied to systems with realistic biological complexity.

## Data Availability

HylleraasMD (HyMD), the HhPF code used to produce all of the results contained in this work is free and openly available, under LGPLv3 licence, at the GitHub repository https://github.com/Cascella-Group-UiO/HyMD. The simulation data supporting the findings reported is openly available at the GitHub repository https://github.com/Cascella-Group-UiO/Publications.

## Supporting Information Available

CG mapping for DPPC, HhPF pressure derivation and implementation, analytic model for biphasic system, details of the Bayesian optimization protocol, and details of constant area simulations (PDF)
