# Supplementary material for: Soft Matter under Pressure: Pushing Particle–Field Molecular Dynamics to the Isobaric Ensemble
Source: J Chem Inf Model. 2023 Mar 28;63(7):2207–17. doi: 10.1021/acs.jcim.3c00186 (PMC10091448; doi:10.1021/acs.jcim.3c00186)
Supplement: Supplementary file 1 — ci3c00186_si_001.pdf [file ci3c00186_si_001.pdf]

Supporting Information for Soft Matter under Pressure: Pushing Particle-Field Molecular Dynamics to the Isobaric Ensemble

Samiran Sen, Morten Ledum, Sigbjørn Løland Bore, and Michele Cascella*

Hylleraas Centre for Quantum Molecular Sciences and Department of Chemistry, University of Oslo, PO Box 1033 Blindern, 0315 Oslo, Norway

E-mail: michele.cascella@kjemi.uio.no

SI: Coarse-grained mapping

For the HhPF simulations run on dipalmitoylphosphatidylcholine (DPPC) lipid bilayers, the coarse-grain (CG) mapping shown in Fig. S1 was used.[1]

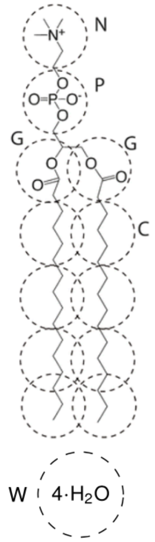

Figure S1: Diagrammatic representation of the coarse-grain mapping used for DPPC and water

SI: Field pressure: A complete derivation

The internal pressure due to a a system of N particles is given by:

$$P_a^{tot} = \frac{2T_a}{\mathcal{V}} - \frac{L_a}{\mathcal{V}} \sum_{i=1}^{N} \frac{\partial \mathcal{U}_{0i}}{\partial L_a} - \frac{L_a}{\mathcal{V}} \frac{\partial W[\{\tilde{\phi}\}]}{\partial L_a} \tag{1}$$

where the first two terms are the standard contributions to the pressure as in any molecular dynamics. All variables are defined in the main text. The third term, call it $P_a^{(3)}$, is the pressure due to the density field part of the Hamiltonian. Here we show the detailed derivation of $P_a^{(3)}$.

$$P_a^{(3)} = -\frac{L_a}{\mathcal{V}} \frac{\partial W[\tilde{\phi}]}{\partial L_a} \tag{2}$$

$$= -\frac{L_a}{\mathcal{V}} \left(\int w(\tilde{\phi}(\mathbf{r})) \frac{\partial d\mathbf{r}}{\partial L_a} + \int \sum_t \frac{\partial w(\tilde{\phi}(\mathbf{r}))}{\partial \tilde{\phi}_t(\mathbf{r})} \frac{\partial \tilde{\phi}_t(\mathbf{r})}{\partial L_a} d\mathbf{r} \right) \tag{3}$$

$$\frac{\partial d\mathbf{r}}{\partial L_a} = \frac{d\mathbf{r}}{L_a} \tag{4}$$

$$\frac{\partial w}{\partial \tilde{\phi}_t(\mathbf{r})} = \overline{V}_t(\mathbf{r})(\text{say}) \tag{5}$$

Putting (4) and (5) into (3):

$$P_a^{(3)} = -\frac{L_a}{\mathcal{V}} \left(\int w(\tilde{\phi}(\mathbf{r})) \frac{d\mathbf{r}}{L_a} + \int \sum_t \overline{V}_t(\mathbf{r}) \frac{\partial \tilde{\phi}_t(\mathbf{r})}{\partial L_a} d\mathbf{r} \right) \tag{6}$$

$$= -\frac{1}{\mathcal{V}} \int w(\tilde{\phi}(\mathbf{r})) d\mathbf{r} - \frac{L_a}{\mathcal{V}} \int \sum_t \overline{V}_t(\mathbf{r}) \frac{\partial \tilde{\phi}_t(\mathbf{r})}{\partial L_a} d\mathbf{r} \tag{7}$$

$$= -\frac{1}{\mathcal{V}} W[\{\tilde{\phi}\}] - \frac{L_a}{\mathcal{V}} \int \sum_t \overline{V}_t(\mathbf{r}) \frac{\partial \tilde{\phi}_t(\mathbf{r})}{\partial L_a} d\mathbf{r} \tag{8}$$

where in the third step, we used the local energy density expression:

$$W[\{\tilde{\phi}\}] = \int w(\{\tilde{\phi}(\mathbf{r})\}) d\mathbf{r} \tag{9}$$

The particle density of type t is defined as:

$$\phi_t(\mathbf{r}) = \sum_{i=1}^{N_t} P(\mathbf{r} - \mathbf{r}_i) \tag{10}$$

which is filtered by the *filter function* $\mathcal{G}$, according to:

$$\tilde{\phi}_t(\mathbf{r}) \equiv \int \phi_t(\mathbf{x})\mathcal{G}(\mathbf{r} - \mathbf{x})\mathrm{d}\mathbf{x} \tag{11}$$

Using the convolution theorem on (11), we obtain in Fourier space:

$$\tilde{\phi}(\mathbf{x}) = \int \hat{\mathcal{G}}(\mathbf{k})\hat{\phi}(\mathbf{k})e^{-i\mathbf{k}\cdot\mathbf{x}}d\mathbf{k} \tag{12}$$

The following (from (13) to (16)) is a list of relations we will use in the simplification of $\partial\tilde{\phi}(\mathbf{x})/\partial L_a$ (from (17) to (25)):

$$\frac{\partial}{\partial L_a}\hat{\phi}(\mathbf{k})e^{-i\mathbf{k}\cdot\mathbf{x}} = 0 \tag{13}$$

$$\frac{\partial d\mathbf{k}}{\partial L_a} = -\frac{1}{L_a}d\mathbf{k} \tag{14}$$

$$\frac{\partial \hat{\mathcal{G}}(\mathbf{k})}{\partial L_a} = \frac{\partial \hat{\mathcal{G}}(\mathbf{k})}{\partial k_a}\frac{\partial k_a}{\partial L_a} \tag{15}$$

$$\frac{\partial \mathbf{k}}{\partial L_a} = -\frac{k_a}{L_a} \tag{16}$$

Note that in (16), the derivative of the wave vector $\mathbf{k}$ with the length of the box in a direction a gives a result in the ath direction only. Using these relations, the derivative becomes:

$$\frac{\partial\tilde{\phi}(\mathbf{x})}{\partial L_a} = \int \frac{\partial\hat{\mathcal{G}}(\mathbf{k})}{\partial L_a}\hat{\phi}(\mathbf{k})e^{-i\mathbf{k}\cdot\mathbf{x}}d\mathbf{k} + \int \hat{\mathcal{G}}(\mathbf{k})\hat{\phi}(\mathbf{k})e^{-i\mathbf{k}\cdot\mathbf{x}}\frac{\partial d\mathbf{k}}{\partial L_a} \tag{17}$$

$$= \int \frac{\partial\hat{\mathcal{G}}(\mathbf{k})}{\partial L_a}\hat{\phi}(\mathbf{k})e^{-i\mathbf{k}\cdot\mathbf{x}}d\mathbf{k} - \frac{1}{L_a}\int \hat{\mathcal{G}}(\mathbf{k})\hat{\phi}(\mathbf{k})e^{-i\mathbf{k}\cdot\mathbf{x}}d\mathbf{k} \tag{18}$$

$$= -\frac{1}{L_a}\left(\int k_a\frac{\partial\hat{\mathcal{G}}(\mathbf{k})}{\partial k_a}\hat{\phi}(\mathbf{k})e^{-i\mathbf{k}\cdot\mathbf{x}}d\mathbf{k} + \int \hat{\mathcal{G}}(\mathbf{k})\hat{\phi}(\mathbf{k})e^{-i\mathbf{k}\cdot\mathbf{x}}d\mathbf{k}\right) \tag{19}$$

$$= -\frac{1}{L_a}\left(\int k_a\frac{\partial\hat{\mathcal{G}}(\mathbf{k})}{\partial k_a}\hat{\phi}(\mathbf{k})e^{-i\mathbf{k}\cdot\mathbf{x}}d\mathbf{k} + \tilde{\phi}(\mathbf{x})\right) \tag{20}$$

Our derivation till this point has been completely general in that we have not specified any functional forms for the energy functional W or the filter $\mathcal{G}$. Since the above expression has a derivative of the filter in Fourier space, it simplifies further if we give $\mathcal{G}$ an explicit form.

We choose $\mathcal{G}$ to be a Gaussian in real space, and by its property, a Gaussian also in Fourier space.

$$\hat{\mathcal{G}}(\mathbf{k}) = e^{-\frac{1}{2}\sigma^2k^2} \tag{21}$$

$$\frac{\partial\hat{\mathcal{G}}(\mathbf{k})}{\partial k_a} = -k_a\sigma^2\hat{\mathcal{G}}(\mathbf{k}) \tag{22}$$

Putting (22) into (20), we get:

$$\frac{\partial\tilde{\phi}(\mathbf{x})}{\partial L_a} = -\frac{1}{L_a}\left[\sigma^2\int(-k_a^2)\hat{\mathcal{G}}(\mathbf{k})\hat{\phi}(\mathbf{k})e^{-i\mathbf{k}\cdot\mathbf{x}}d\mathbf{k} + \tilde{\phi}(\mathbf{x})\right] \tag{23}$$

$$= -\frac{1}{L_a}\left(\sigma^2\int(-k_a^2)\hat{\mathcal{G}}(\mathbf{k})\hat{\phi}(\mathbf{k})e^{-i\mathbf{k}\cdot\mathbf{x}}d\mathbf{k} + \tilde{\phi}(\mathbf{x})\right) \tag{24}$$

$$= -\frac{\sigma^2}{L_a}\nabla_a^2\tilde{\phi}(\mathbf{x}) - \frac{\tilde{\phi}(\mathbf{x})}{L_a} \tag{25}$$

Putting (25) into (8), with $\tilde{\phi}$ as $\tilde{\phi}_t$ we get:

$$P_a^{(3)} = \frac{1}{\mathcal{V}}\left[-W[\{\tilde{\phi}(\mathbf{r})\}] + \int \sum_t \overline{V}_t(\mathbf{r})\tilde{\phi}_t(\mathbf{r})d\mathbf{r} + \int \sum_t \sigma^2 \overline{V}_t(\mathbf{r})\nabla_a^2 \tilde{\phi}_t(\mathbf{r})d\mathbf{r}\right] \tag{26}$$

SI: Pressure implementation

Laplacian field pressure term: The additional computational costs for NPT simulations are associated with the third term of (26), involving the Laplacian of the density fields. We use highly parallel Fast Fourier Transform (FFT) to evaluate the Laplacian as follows:

$$\begin{aligned} &\tilde{\phi}(\mathbf{r}) \\ &\rightarrow \tilde{\phi}(\mathbf{k}) = FFT\left(\tilde{\phi}(\mathbf{r})\right) \\ &\rightarrow \nabla_a^2 \tilde{\phi}(\mathbf{r}) = FFT^{-1}\left(-|\mathbf{k}|^2 \tilde{\phi}(\mathbf{k})\right) \end{aligned} \tag{27}$$

Parallelization of pressure calculations: The HylleraasMD (HyMD) code uses MPI parallelization with the PFFT backend for performing Fourier transforms. We employ the scheme of *pencil* decomposition for good scalability for large systems. For this, the system is divided into many small units and the computation of pressure is massively compartmentalized into different MPI ranks. For example, a system with 20000 particles can be usually divided among 300 cores so pressure calculations are enormously parallel, greatly cutting down the computation time.

Local pressure calculations: The current implementation of NPT runs computes and stores by default the total field pressure in each Cartesian direction. In order to investigate the local pressure variation across the simulation box, it is possible to straightforwardly skip the step of summing the local pressure contributions across the simulation box and instead write out all grid values into the trajectory.

Barostat: We used the Berendsen barostat (ref. 38 in main text) with an isotropic coupling in case of homogeneous media, and semiisotropic coupling in case of interfacial systems (binary mixtures, membranes). To accelerate the process of equilbration, a strong pressure coupling constant is often used (small τ_P), which is later released during MD when collecting equilibrium data

for analysis. The simulation box and coordinates is rescaled every n_b steps according to:

$$\left\{L_{L,(n+n_b)},\, \mathbf{r}_{L,(n+n_b)}\right\} = \alpha_L^{\frac{1}{3}} \left\{L_{L,n}\, \mathbf{r}_{L,(n+n_b-1)}\right\} \tag{28}$$

$$\left\{L_{N,(n+n_b)},\, \mathbf{r}_{N,(n+n_b)}\right\} = \alpha_N^{\frac{1}{3}} \left\{L_{N,n}\, \mathbf{r}_{N,(n+n_b-1)}\right\} \tag{29}$$

$$\alpha_{L,N} = 1 - \frac{n_b \Delta t}{\tau_P} \beta \left(P^t_{L,N} - P_{L,N}\right) \tag{30}$$

where L_L and L_N are the lengths of the box along and normal to the membrane plane, respectively, $\mathbf{r}$ is the position of each particle, Δt is the time-step, $\beta = 4.6 \times 10^{-5}$ bar^{-1} is the isothermal compressibility of water at NTP, $P^t_{L,N}$ and $P_{L,N}$ are the target and current internal pressures, respectively. In case of membrane systems, the isotropy is conventionally supposed in the x–y direction (L) and the z-direction (N) is treated independently.

Barostat call frequency: HyMD uses a multiple timestep scheme according to the reversible reference system propagator algorithm (rRESPA)[2] where slow varying field-forces are updated less frequently (every outer rRESPA interval) than the intramolecular forces due to bonds (every inner rRESPA interval). It was tested that the barostat can be called every $n_b = 10$ (outer rRESPA) steps without causing artifacts while significantly increasing the speed of the code. In a benchmark study of 528 DPPC lipids in a $12.96 \times 12.96 \times 14.44$ nm box, the speed of an NPT run running in 32 parallel cores for $n_b = 10$ was found to be 2.4 times more than for $n_b = 1$. It is however advised to use a small n_b until the system has reached equilibrium.

SI: Analytic study of biphasic system

We can split the filtered potential $\overline{V}_t(\mathbf{r})$ for particle type t:

$$\overline{V}_t(\{\tilde{\phi}_i\}) = \frac{\partial w(\{\tilde{\phi}_i\})}{\partial \tilde{\phi}_t} \tag{31}$$

into two parts - an interaction $\overline{V}_{\chi,t}$ and a compressibility $\overline{V}_\kappa$ filtered potential respectively:

$$\overline{V}_t(\{\tilde{\phi}_i\}) = \overline{V}_{\chi,t}(\{\tilde{\phi}_i\}) + \overline{V}_\kappa(\{\tilde{\phi}_i\}) \tag{32}$$

$$\overline{V}_{\chi,t}(\{\tilde{\phi}_i\}) = \frac{1}{\rho_0}\sum_i \chi_{it}\tilde{\phi}_i \tag{33}$$

$$\overline{V}_\kappa(\{\tilde{\phi}_i\}) = \frac{1}{\kappa\rho_0}(\sum_i \tilde{\phi}_i - a) \tag{34}$$

A more comprehensive expansion of $\overline{V}$ is given in SI: Expansion of the filtered potential. To model a simple phase-separating toy model, consider a simple binary system of particles of type A and B that repel each other. We illustrate through a simple function the importance of the anisotropic term in our pressure equation (26). The binary system may be analytically studied using a sigmoidal function $\tilde{\phi}(z)$ of the following form with different parameters for types A and B:

$$\tilde{\phi}(z) = \frac{y_m}{1 + e^{-a(z-b)}} \tag{35}$$

$$A : a = -2;\ b = 6;\ y_m = 1 \tag{36}$$

$$B : a = \ \ 2;\ b = 6;\ y_m = 1 \tag{37}$$

Fig. 2A in the main text shows the density and derivatives of these functions. The two plots in the bottom row are proportional to the interaction part of the third term in (26). See that the contribution from (34) is a constant that can merely shift the curve vertically and does not affect its nature, so we disregard it in this analysis. The significance is minimal in bulk (where it is zero) and high at the interfacial region (where it is large and positive). This produces a driving force on the system in a direction perpendicular to the interfacial plane, causing it to expand normally

and shrink laterally. This consequence is of immense significance. Driven by surface tension, one component of the binary mixture tries to minimize its area of contact with the other component when put together.

We built such a biphasic system in our scheme of HhPF where a large positive interaction parameter $\tilde{\chi} = 36$ defined the phase-separating nature of A and B. We subjected it to a constant external pressure of 1 bar. Fig. 2B in the main text shows the formation of a capillary as expected.

SI: Machine learning with Bayesian Optimization

We used the same protocol of Bayesian Optimization (BO) with the UCB acquisition function to find HhPF $\tilde{\chi}$ interaction parameters which recover all-atom structural properties of the DPPC bilayer as reported by Ledum *et al.* (ref. 42 in main text) The choice of this method is justified by its strong convergence when coupled with an upper confidence bound (UCB) acquisition function (ref. 55 in main text). We used the same kernel, namely, the Matérn covariance kernel with smoothing parameter $\nu = 5/2$ and a diagonal white kernel accounting for noisy sampling.[3,4] We minimised the loss function targeting the area per lipid and AA lateral density profiles.

Even though BO, unlike most other ML schemes, offers information about how confident the optimizer is about any given parameter set, it is still hard to interpret the physics of model parameters subsequent to optimizing them. Like the previous application of this approach, we found a large range of applicability for the parameters for which the reference bilayer organization was well reproduced, with many local minima in somewhat diverse regions of the parameter space. After optimization, we selected 15 parameter sets with the lowest SMAPE. While most of these sets produced numerically consistent density profiles and area compressibilities, we shortlisted two sets of $\tilde{\chi}$-parameters that made the most physical sense. These are listed in Table S1 alongwith the area per lipid (A_L) and area compressibility ($\overline{\mathrm{K}}$) they produce.

Previously optimized $\tilde{\chi}$-parameteres for DPPC solely on the basis of fitting the density profiles are listed in ref. 42 in main text. As finding optimal DPPC parameters for the HhPF model is in no way the focus of this work, we leave scrutiny of the numerical values of the parameters and their physical interpretations to future work. We must state at this point that membrane bilayer models in hPF in the past (ref. 30 in main text) have been extremely rigid (two orders of magnitude higher area compressibility) and our values are much closer to experiment.

Table S1: Shortlised set of $\tilde{\chi}$-parameters used in the NPT simulations for DPPC bilayers. All $\tilde{\chi}$ values are in $\mathrm{kJ\,mol^{-1}}$, A_L in $\mathrm{nm^2}$, $\overline{\mathrm{K}}$ in $\mathrm{mN\,m^{-1}}$

Set	$\tilde{\chi}$										SMAPE	A_L	$\overline{K}$
	N,P	N,G	N,C	N,W	P,G	P,C	P,W	G,C	G,W	C,W			
1	-10.41	-10.56	-13.73	-15.51	-8.62	19.96	-3.02	16.46	-12.05	24.28	18.75	0.630	215
2	-4.65	-10.46	-5.01	-18.69	-8.76	17.57	-7.25	10.03	-2.25	17.40	19.04	0.646	145

SI: Constant area simulations

For each set of $\tilde{\chi}$-parameters that were NPT-optimised using BO, we ran NPT simulations in the following manner:

1. Relax initial setup
   (a) Set target lateral pressure P_L^t and normal pressure P_N^t to 1 bar.
   (b) NPT run for 20 ns.
   (c) Extract last configuration which ensures a completely relaxed system. In our case, the system (DPPC2_A) reaches equilibrium in approximately 5 ns.
2. Subject the system to lateral tension
   (a) Set $P_L^t = -50$ bar and $P_L^t = 1$ bar. Note that a large negative P_L^t can break the membrane.
   (b) NPT run for 30 ns.
   (c) Extract them every 1 ns i.e. at different lateral tensions.
3. Constant area runs
   (a) For each extracted configuration corresponding to a different lateral tension i.e. different area per lipid A, NPT run with $P_L^t =$ False i.e. barostat switched off in the lateral direction and $P_N^t = 1$ bar.
   (b) Calculate average surface tension $\langle\gamma\rangle$ for each run.
   (c) Plot A against $\langle\gamma\rangle$ and using a least square fit, obtain the slope $d\langle\gamma\rangle/dA$. Multiply it with area per lipid from the configuration at zero tension, A_{L_0}, to obtain the area compressibility $\overline{K}$.

Fig. S2 shows two best representative cases corresponding to sets 1 and 2 of Table S1 for which the area compressibility obtained was 225 and 162 mN m^{-1} respectively. From literature (ref. 59-62 in main text), we know a DPPC bilayer exhibits an area compressibility of around $230-350$ mN m^{-1}

with which our results were in excellent agreement. For comparison, previous hPF studies have reported very rigid membranes with area compressibilities of 22000 mN m^{-1} (ref. 30 in main text).

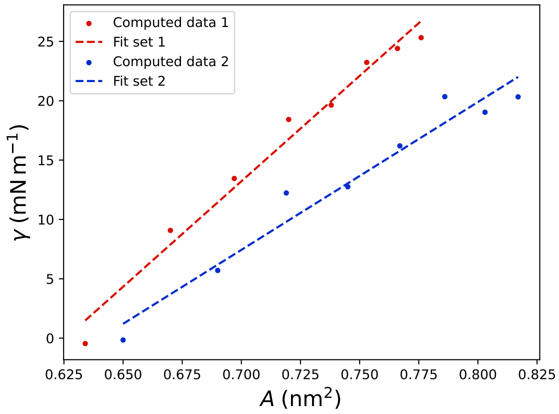

Figure S2: Surface tension measured by varying the lateral stretching force on the membrane. The two lines shown are for two different sets of machine-learnt $\tilde{\chi}$-parameters.

SI: Expansion of the filtered potential

The following is a detailed mathematical expansion of the filtered potential used in HhPF pressure calculations for a particle of type t.

$$\overline{V}_t(\{\tilde{\phi}_i\},\tilde{\phi}_t) = \frac{\partial w(\{\tilde{\phi}_i\})}{\partial \tilde{\phi}_t} \tag{38}$$

$$= \frac{\partial w_{\tilde{\chi}}(\{\tilde{\phi}_i\})}{\partial \tilde{\phi}_t} + \frac{\partial w_{\kappa}(\{\tilde{\phi}_i\})}{\partial \tilde{\phi}_t} \tag{39}$$

$$= \overline{V}_{\tilde{\chi},t}(\{\tilde{\phi}_i\},\tilde{\phi}_t) + \overline{V}_{\kappa}(\{\tilde{\phi}_i\},\tilde{\phi}_t) \tag{40}$$

$$\overline{V}_{\tilde{\chi},t} = \frac{1}{\rho_0}\sum_{i<j}\tilde{\chi}_{ij}\frac{\partial(\tilde{\phi}_i\tilde{\phi}_j)}{\partial \tilde{\phi}_t} \tag{41}$$

$$= \frac{1}{2\rho_0}\sum_{i,j}\tilde{\chi}_{ij}(\tilde{\phi}_i\delta_{tj} + \tilde{\phi}_j\delta_{ti}) \tag{42}$$

$$= \frac{1}{\rho_0}\sum_{i}\tilde{\chi}_{it}\tilde{\phi}_i \tag{43}$$

$$\overline{V}_{\kappa} = \frac{1}{\kappa\rho_0}(\sum_{i}\tilde{\phi}_i - a) \tag{44}$$

δ_{ij} is the Kronecker delta. Notice that there is no subscript t for the incompressibility term because it is independent of the type of particle.

References

(S1) J. Marrink, S.; Jelger Risselada, H.; Yefimov, S.; Peter Tieleman, D.; de Vries, H. A. The MARTINI Force Field: Coarse Grained Model for Biomolecular Simulations. *J. Phys. Chem. B* . **2007**, *111*, 7812–7824.

(S2) Tuckerman, M.; Berne, B. J.; Martyna, G. J. Reversible multiple time scale molecular dynamics. *J. Chem. Phys.* **1992**, *97*, 1990–2001.

(S3) Matérn, B. *Spatial Variations*; Springer: New york, 1986.

(S4) Stein, M. *Interpolation of Spatial Data: Some Theory for Kriging. Springer Science & Business Media*; 2012.
